# Biodegradable composites from organic waste as a circular solution for improving soil fertility, water retention, and plant productivity

**DOI:** 10.1038/s41598-026-43468-x

**Published:** 2026-03-18

**Authors:** Daria Marczak, Krzysztof Lejcuś, Grzegorz Kulczycki, Malcolm J. Hawkesford

**Affiliations:** 1 Institute of Environmental Engineering, Wrocław University of Environmental and Life Sciences, Wrocław, 50-363 Poland; 2 Department of Plant Nutrition, Wrocław University of Environmental and Life Sciences, Wrocław, 50-363 Poland; 3 Sustainable Soils and Crops Department, Rothamsted Research, Harpenden, Hertfordshire UK

**Keywords:** Biodegradable composites, Biochar, Soil fertility, Soil amendments, Nutrient availability, Trichoderma, Ecology, Ecology, Environmental sciences, Plant sciences

## Abstract

Growing environmental problems and challenges related to plant production require the use of innovative soil additives that reduce water deficits and soil fertility decline while caring for the environment. This study presents a sustainable approach to improving soil properties and plant growth conditions through the use of innovative biodegradable soil additives made from organic waste with the addition of biochar and Trichoderma. A two-year field experiment conducted in difficult habitats included an assessment of the impact biodegradable composites on soil and plants properties, water availability. The use of composites resulted in intensive plant growth, increasing biomass gains by up to 190%. A significant improvement in soil parameters was noted, as evidenced by increases of up to 119%, 177%, and 145% in the N, P, K content of the soil. The amendments also enhanced plant water status, as evidenced by a 20% increase in leaf relative water content, and the beneficial effects persisted into the second year of the study. The results obtained indicate that the combination of biodegradable fibers with biochar has significant potential in the creation of circular environmental technologies. The research provides new insights into the dynamics of natural fiber biodegradation and the functional role of biochar in improving soil quality.

## Introduction

### Global environmental problems

Human activities, environmental pollution, soil degradation and climate changes are increasingly threatening the achievement of sustainable development goals^1^. The intensifying demand for natural resources leads to an increasing pressure on the environment and has a negative impact on ecosystems around the globe^2^. In recent years, there has been a dynamic increase in the frequency and extent of dry periods, leading to increasingly frequent occurrence of droughts and a deepening of their consequences^3^. Plant cultivation, including agriculture, horticulture and environmental engineering applications, is among the sectors characterised by high water demand and may contribute to increasing water deficits and environmental quality deterioration, depending on management practices and local conditions^4,5^.

Regular, well-planned irrigation is crucial for sustainable crop cultivation, especially under future climate uncertainties. Increasing droughts and soil degradation threaten global food security^6^. Soils, essential for nutrient cycling, water retention, carbon sequestration, and food production, require careful management^7,8^. With the population expected to reach 10 billion by 2050, plant production must rise by up to 50%^9^. However, intensification based on fertilizers and pesticides risks soil degradation, water pollution, biodiversity loss, and higher CO_2_ emissions, while rising fertilizer costs add economic pressure^10^.

Soil degradation is a major global problem, encompassing a range of physical, chemical, and biological processes, including erosion, loss of organic matter, soil compaction, salinization, and contamination, with 70% of soils in the EU in poor condition^11,12^. These processes often interact and reinforce one another, accelerating soil decline and limiting ecosystem services, particularly under intensive land use and climate change pressure^13,14^. Erosion by water and wind is especially concerning, as its rate is about 1.5 times higher than natural soil formation^15^. The issue is hard to quantify, and awareness remains low^16^. Key drivers include land geomorphology, extreme weather, and human activity (e.g., land use changes, loss of plant cover)^17^. Erosion depletes fertile topsoil, organic matter, and water retention, lowering soil productivity and increasing plant stress^11,18,19^. Permanent vegetation cover (grass, shrubs, trees) is an effective countermeasure, as it protects soil, enhances structure, and reduces runoff, though establishing plants in degraded areas remains challenging^20,21^.

### Alternative environmental technologies

There are many available methods that enable effective introduction and maintenance of vegetation, whether in agriculture, horticulture, environmental engineering, or in difficult habitats. The main methods are geotextiles, composites, and soil conditioners. Geotextiles are commonly applied as an element of anti-erosion reinforcement and protection, as protection from weeds and pests, and as a separation or thermal insulation layer^22–24^. Most are plastic-based and non-degradable, contributing to pollution, with global fibre use projected to reach 150 million tonnes by 2050^25^. Composites based on natural fibres (of plant and/or animal origin) are becoming increasingly important in environmental applications^26^. Natural fibres, such as wool, jute, flax, wood, bamboo or hemp are characterised by desirable properties in terms of the production of sustainable technologies^27^. The main properties of natural fibres include high mechanical resistance during use, elasticity, hygroscopicity, biodegradability over time under natural conditions, ease of processing, and low acquisition costs^28,29^. Moreover, these fibres are often readily available as by-products or residual materials from agricultural and industrial processing, and their reuse can therefore contribute to reducing the amount of unused biomass and supporting circular economy strategies.

Natural fibres are mainly used in erosion mats, nets, ropes, insulation, and vegetation mats^30,31^. Their biodegradability and ease of processing make them promising for multi-component composites, such as the biodegradable water absorbing geocomposite (BioWAG), which combines nonwoven fabric, a superabsorbent (SAP), and a skeleton^32^. While effective in supplying water and nutrients, SAPs require complex chemical production and pose environmental concerns^33^. Therefore, research increasingly explores natural alternatives, such as biochar from biomass pyrolysis, which improves soil water retention, fertility, and carbon sequestration while reducing greenhouse gas emissions^34,35^.

###  Development of the new technology

In view of the environmental problems presented above, one of the main challenges is the development of sustainable technologies that will improve the efficiency of plant production while minimising the negative impact on the environment^6^. The challenges posed by climate change point even more clearly to the need to develop technologies that will enable adaptation to climate changes and reasonable soil fertility management. These technologies should simultaneously take into account the socio−economic barriers. Reducing the costs related to the production of innovative environmental technologies by applying the appropriate waste materials might make plant production more sustainable and reliable^36,37^.

To confront these challenges, the authors of the present study have designed and tested in field conditions composites consisting of waste materials, including: waste fibres of plant and animal origin, biochar manufactured from plant waste, and a preparation based on fungi from the Trichoderma genus. This technology is designed to provide sustainable support for the vegetation of plants for example as elements of biotechnological reinforcement of earthwork objects, but also in other environmental applications.

The composite developed is based on the synergy of three elements: biodegradable fibers, biochar, and Trichoderma fungi. Natural fibers act as a structural carrier and release nutrients during biodegradation, supporting plant growth^38–40^. Thanks to its high porosity, biochar increases water and mineral ion retention, improves soil quality, and sequesters carbon^41–43^. Trichoderma-based microbial preparations stimulate plant growth, limit the development of pathogens, and increase the availability of minerals^44,45^.

The combination of these three components results in a material that simultaneously stabilizes the soil, improves its chemical properties, and supports biological processes^46,47^. Unlike widely used synthetic polymer or SAP composites, the proposed solution is fully biodegradable and based on waste materials. Advances in research in this area point to the growing importance of multi-component biocomposites as innovative technologies supporting sustainable plant production and soil remediation^48,49^.

###  Objective and scope of the study

This study aimed to develop sustainable soil conditioners from organic waste materials of plant and animal origin. The main objectives were: (1) Assess the potential of selected organic waste types for soil conditioner production. (2) Evaluate nonwoven composite properties under actual field conditions. (3) Determine composite effects on grass species vegetation. To achieve these objectives, we conducted a two−year field experiment on a test embankment. Applying this sustainable technology under field conditions enabled us to understand complex environmental processes and determine the technology’s practical potential. The results are relevant for scientists, practitioners, and decision−makers seeking alternative solutions for vegetation establishment in challenging environments.

Based on the literature review and the documented functional properties of natural waste fibres and biocarbon, we hypothesised that biodegradable composite materials would promote plant growth primarily through the reduction of soil moisture loss, enhancement of soil water retention capacity, and improvement of nutrient availability within the root zone when compared with unamended soil. Moreover, we postulated that the incorporation of biocarbon would stabilise and prolong these beneficial effects by limiting nutrient leaching and further increasing water retention, thereby sustaining system performance and ensuring long−term effectiveness for a minimum of two consecutive growing seasons.

## Results

### Fresh and dry weight of grass

The field research demonstrated that the grass growing on sites with biodegradable composites was in a significantly better condition than that on control sites (without additives). These observations were confirmed, among others, by the results of fresh and dry weight of plants. During the first and second vegetation season, the results from three swathes were analysed (i.e. 6 swathes in total). For both vegetation seasons, the vegetation growth in the fresh and dry weight of grass on all sites with installed biodegradable composites remained significantly higher than on the control sites. The highest effectiveness of the new soil conditioner was noted in the first vegetation season, during which the increases in fresh and dry weight of plants were 80 to 190% higher than those on the respective control fields (Table 1). In the second vegetation season, the increases in fresh and dry weight were 44 to 123% higher than on the respective control sites. In the first year of the experiment, variants enriched by nonwoven from 100% wool (A1; A1 + BIOC; A1 + BIOC+TRICH) proved to be the most effective. On the other hand, in the subsequent season better results were noted for variants based on the nonwoven consisting of a mix of wool ad jute (B1; B1 + BIOC; B1 + BIOC+TRICH).

**Table 1:** Fresh and dry matter yield of grass in the years 2023–2024. Values indicated by the same letter are not significantly different (a = 0.05).

Treatment	2023		2024	
	Fresh matter	Dry matter	Fresh matter	Dry matter
	g∙m^−2^			
K	149.97b	25.50b	138.17d	19.51c
B1	272.51ab	47.32ab	207.93c	29.84b
B1+BIOC	326.15a	56.18ab	299.65a	43.43a
B1+BIOC+TRICH	350.32a	60.72a	297.16ab	42.90a
A1	336.68a	57.48ab	198.36c	28.78b
A1+BIOC	421.89a	73.49a	260.38b	37.22a
A1+BIOC+TRICH	426.56a	73.56a	260.02b	37.27a

### Relative water content (RWC)

**Fig. 1:**
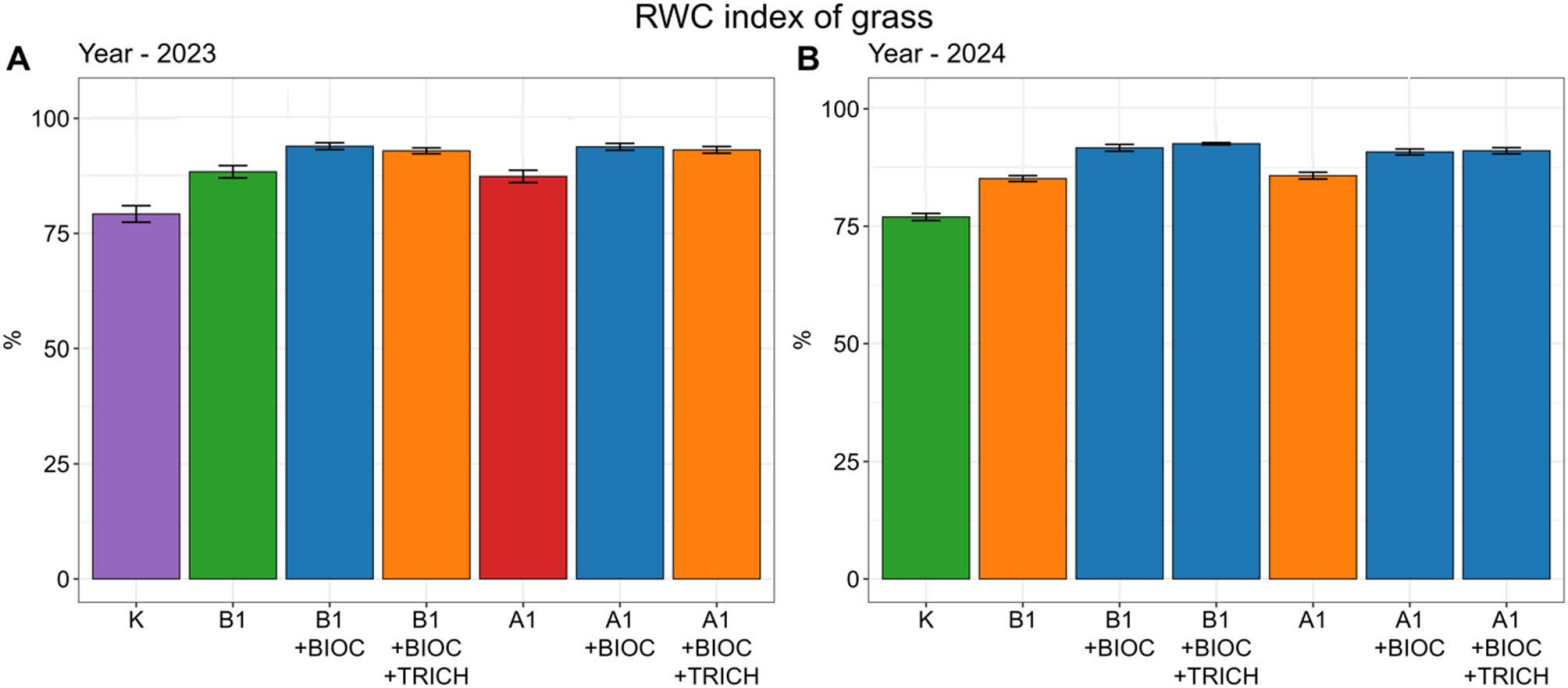
RWC index in the years 2023 and 2024. Errors refer to the standard deviation error (SE); values marked with the same letter do not differ significantly (a = 0.05).

The application of biodegradable composites had a beneficial effect on the values of the RWC index. On sites with composites, the RWC was 10–19% higher in the first season, and 11–20% higher in the second season (Fig. 1). The best results were noted on sites with the addition of biochar, i.e.: A1 + BIOC; A1 + BIOC+TRICH; B1 + BIOC; B1 + BIOC+TRICH.

### Chemical composition of plants

The application of composites had a significant influence on the content of the selected micro- and macro-elements in plants (Table 2). In the first vegetation season, on sites with composites based on wool and jute (B1, B1 + BIOC, B1 + BIOC+TRICH), the content of N was higher by 64 to 94%, the content of P was 47 to 53% higher, and the content of K was 6 to 11% higher. In the second vegetation season, the content of N was higher by 29 to 41%, P by 28 to 36%, and K by 34 to 49%.

For the variant with the 100% wool nonwoven (A1, A1 + BIOC, A1 + BIOC+TRICH), the content of N was 59 to 93% higher, the content of P was 26 to 73% higher, and the content of K was 11 to 37% higher in the first vegetation season. During the second season, the accumulation of N was 21 to 32% higher, of P 21 to 37% higher, and of K 24 to 41% higher.

**Table 2:** Content of selected macro- and microelements in plant material in 2023 and 2024. Values indicated by the same letter are not significantly different (a = 0.05).

Treatment	N	P	K	S	Mn
	g kg^−1^ DM				
2023					
K	22.49d	3.05d	34.33d	2.85e	21.03d
B1	43.56a	4.64b	37.83c	4.34bc	33.65b
B1+BIOC	36.90bc	4.68b	38.13c	4.12d	34.65b
B1+BIOC+TRICH	38.80b	4.49b	36.54c	4.22cd	35.43b
A1	43.31a	5.28a	47.04a	4.67a	24.05c
A1+BIOC	38.68b	4.76b	43.32b	4.34bc	24.47c
A1+BIOC+TRICH	35.83c	3.85c	37.95c	4.38b	39.73a
2024					
K	20.91d	2.87d	23.43e	1.73d	21.63d
B1	26.90b	3.67bc	31.36c	2.78c	27.25c
B1+BIOC	29.42a	3.90a	34.92a	3.14ab	31.58ab
B1+BIOC+TRICH	27.74b	3.76ab	34.63a	3.24a	32.18a
A1	25.22c	3.47c	28.95d	2.85bc	25.83c
A1+BIOC	27.19b	3.80ab	32.03bc	3.14ab	29.88b
A1+BIOC+TRICH	27.60b	3.92a	32.95bc	3.02ab	32.35a

### Root system

**Fig. 2:**
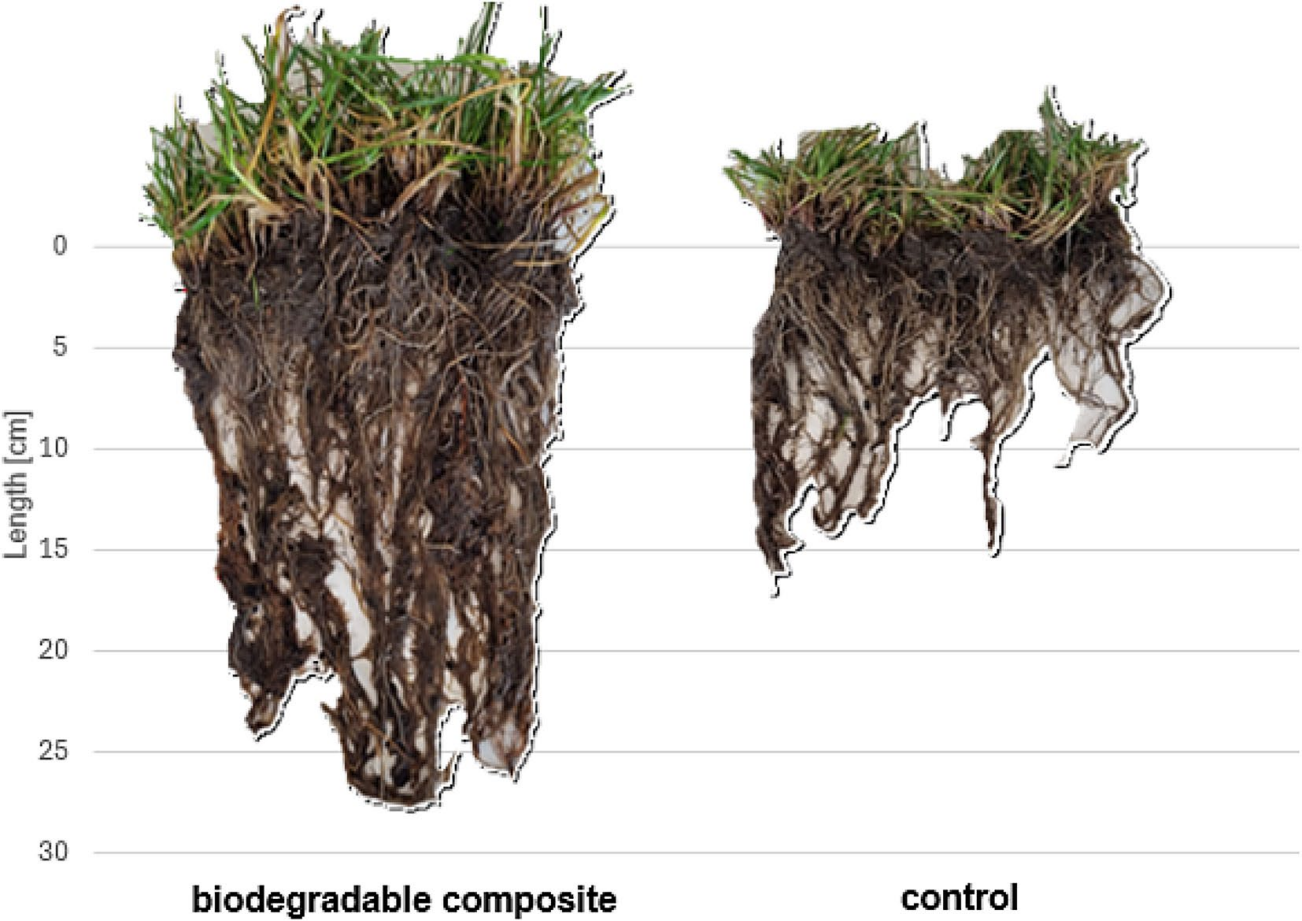
Effect of biodegradable composites on grass root system growth compared to control.

The presence of nonwoven composites provided conditions that fostered the development of the root system of grass. The Root Dry Mass Density (RDMD) index on sites with composites was, on average, 71 to 119% higher in the first vegetation season, and 42 to 87% higher in the second season. Significant differences in Root Length Density RLD were also noted; the index was 29 to 56% higher in the first season and 20 to 36% higher during the second season (Table 3; Fig. 2).

**Table 3:** Average values of the biometric parameters of the root system in the years 2023−2024. Values indicated by the same letter are not significantly different (a = 0.05).

Treatment	2023		2024	
	RLD	RDMD	RLD	RDMD
	m m^−3^	g m^−3^	m m^−3^	g m^−3^
K	65.63c	433.33c	72.92b	501.04c
B1	84.90b	739.58b	87.50a	709.38b
B1+BIOC	98.96a	833.33ab	95.83a	891.67a
B1+BIOC+TRICH	102.08a	876.04a	97.92a	907.29a
A1	94.27ab	743.75b	88.54a	713.54b
A1+BIOC	100.00a	926.04a	95.83a	938.54a
A1+BIOC+TRICH	102.08a	951.04a	98.96a	934.38a

### Soil properties

In the first and second vegetation seasons, the values of pH on all analysed sites remained on the same level of approximately ± 7 pH, which means that the pH of soils was neutral (Table 4). The soil on control sites was not very rich in nutrients such as P, K, N. The application of nonwoven composites resulted in significant increases in the content of selected macro- and microelements in the soil, which are commonly used as indicators of improved nutrient status. The largest differences were noted during the first vegetation season. During that time, the concentration of N in the soil on sites with installed nonwoven composites was 78 to 119% higher than on the control sites. As for P, the differences ranged from 107 to 176%, and for K from 95 to 146%. Significant differences were also observed for S (values higher by 28−48%), Mn (values higher by 7–30%).

In the subsequent vegetation season, the content of the selected elements remained on a significantly higher level. For N, the concentrations noted on sites with nonwoven composites were 24−46% higher, for P the values were 48−92% higher, and for K the differences ranged from 35−75%. Significant differences were noted for the other elements: for S 23−38% higher, and for Mn they were 16−24% higher. Notably, in the first season the best results were observed on sites with installed composites based on 100% wool nonwoven, i.e. A1, A1 + BIOC, A1 + BIOC+TRICH. On the other hand, in the subsequent season, better results were noted on sites with installed composites based on the nonwoven consisting in 80% of waste wool and in 20% of waste jute, i.e. B1, B1 + BIOC, B1 + BIOC+TRICH.

**Table 4:** Soil parameters noted in the years 2023−2024. Values indicated by the same letter are not significantly different (a = 0.05).

Treatment	**2023**						
	**pH**	**C**	**N**	**S**	**P**	**K**	**Mn**
	1 M KCl dm^−3^	%		g kg^−1^ soil	mg 100g^−1^ soil		mg kg^−1^ soil
K	6.90c	1.11b	0.086c	0.494e	7.42e	6.29d	95.26c
B1	7.01b	1.40ab	0.188a	0.646cd	16.35c	13.11b	101.77b
B1+BIOC	7.07ab	1.61a	0.153b	0.630d	15.38d	12.30c	102.00b
B1+BIOC+TRICH	7.14a	1.58a	0.161ab	0.639cd	16.43c	12.36c	101.54b
A1	7.06ab	1.59a	0.189a	0.731a	20.51a	15.46a	120.09a
A1+BIOC	7.12a	1.42ab	0.172ab	0.652c	18.97b	15.27a	123.77a
A1+BIOC+TRICH	7.13a	1.45ab	0.176ab	0.686b	19.32b	15.15a	120.99a
K	6.94c	1.27e	0.109c	0.398d	7.10d	5.95d	58.81c
2024							
B1	7.02b	1.57d	0.147ab	0.505bc	10.50c	8.48c	72.85a
B1+BIOC	7.14a	2.02b	0.159a	0.532ab	12.52ab	10.15a	71.42a
B1+BIOC+TRICH	7.16a	2.13ab	0.156a	0.550a	13.66a	10.43a	72.30a
A1	6.99bc	1.69c	0.135b	0.488c	10.86c	8.05c	68.35b
A1+BIOC	7.15a	2.14a	0.158a	0.550a	12.15b	9.22b	71.33a
A1+BIOC+TRICH	7.15a	2.10ab	0.157a	0.543a	12.38b	9.45b	72.43a

## Discussion

Sustainable development goals emphasise the need to take a holistic approach to plant cultivation, environmental protection, and taking actions for the climate by rationally improving soil fertility and limiting water consumption^50^. Moreover, multiple strategies were developed (e.g. the Green Deal) with the objectives that include the protection of soils while at the same time reducing the consumption of raw materials and generation of waste^51^. These objectives may be achieved by the search for and implementation of effective innovative solutions, as confirmed by laboratory and field tests. Based on the results presented here, the nonwoven composites produced from waste fibres constitute one such solution.

Organic waste represents a rational raw material for the production of biodegradable soil additives. In this study, low-quality wool, a by product of sheep farming, as well as wool and jute waste obtained from local textile factories involved in clothing processing in Poland, were used to produce biodegradable nonwovens. These materials are considered difficult to manage waste streams. Biochar was obtained from plant waste, miscanthus, commonly used as an energy crop. The use of this type of waste is in line with current challenges associated with the circular economy. As reported by Miatto et al^52^., the agricultural sector still fails to fully exploit the potential for rational biomass use, despite the wide range of applications organic waste could offer in supporting crop production and other environmental technologies.

Europe has significant raw material potential in terms of wool and other natural fibres. According to Eurostat data from 2024, the sheep population in the European Union was approximately 57 million, with countries such as Spain, Romania, and Greece having the largest sheep populations: Spain accounting for nearly 24% of the EU total and Romania for approximately 18% (source: Eurostat, https://ec.europa.eu/eurostat). At the same time, the global economy faces a serious problem related to textile waste, with more than 85% of this type of waste ending up in landfills or being incinerated, thereby contributing to environmental pollution^53^. Incorporating wool and jute waste, as well as other organic residues, into the production cycle not only reduces disposal costs but also shortens supply chains, decreases energy consumption and pollutant emissions, and enables the creation of functional products that support soil fertility and water retention. Furthermore, in the face of growing competition in the production of environmentally friendly solutions, such materials can become an important element of sustainable resource management strategies in the agricultural and environmental sectors.

### Increase in the fresh and dry weight of plants

The increase in the fresh and dry weight of plants confirmed that the developed nonwoven composite was highly effective in supporting the vegetation of selected plant species. The plants growing on all sites with composites developed more intensely than on control sites, as shown by the increases in the fresh weight of grass, which were up to 190% higher. The composites led to an intensive development of the root system and improved anchoring of grass, even in the adverse conditions on the slope. Such effectiveness of the solution likely resulted from combining two materials of complementary properties. Non-woven fabric made from plant and animal fibres undergoes biodegradation in the soil, leading to the decomposition of compounds that are naturally rich in nitrogen and sulphur, among other things^54,55^. These processes cause the gradual release of nutrients into the soil, which has been repeatedly confirmed in studies on the biodegradation of wool and plant fibres used as fertilisers or soil additives^56,57^.

At the same time, biochar obtained from plant biomass improved soil properties by increasing its retention capacity, which promoted the retention of water and nutrients in the root zone^58^. Numerous studies have shown that biochar reduces nitrogen and phosphorus leaching and increases their availability to plants^59^. As a result, the combination of nonwoven fabric and biochar created an environment conducive to supplying plants with water and nutrients.

The results recorded in two consecutive growing seasons confirm the positive effect of the developed nonwoven composites on plant fresh and dry weight gains. However, as the additives were made of biodegradable materials, there will be a gradual reduction of their effectiveness over time. In the first year, the additives were characterized by maximum effectiveness, which translated into the highest increases in plant fresh dry weight. In the following year, there was a natural decline in the effectiveness of this technology, which was due to the gradual biodegradation of the composites in the soil. It is noteworthy, however, that the difference in growth between the control and composite-enriched sites was still significant. The effectiveness of the solution may also have been affected by weather conditions. In the first year of the experiment (2023), the initial phase of plant growth had slightly higher precipitation totals (May-July) with lower temperatures, which may have favoured vegetation growth. On the other hand, the water shortage recorded in September 2023, may have limited plant growth in the second half of the growing season. In the second year of observation (2024), precipitation was slightly lower in the first half of the year, but higher in the second. This may also have affected the distribution of grass growth throughout the year.

Findings that pointed to the positive influence of plant and animal fibres have been discussed in many publications. Broda et al^60^. conducted a field experiment with wool fibre and noted a significant increase in the yield of wheat. In that case, a correlation was noticed: the higher the share of wool, the higher were also the crop yields. In the first vegetation season, with a 2% wool content, the crops were 3 times higher yielding than on the control site (without additives). Moreover, the crop yields from the same plot were also 20% higher than from the field where mineral fertilizer alone was applied. Other research by Broda et al^61,62^. clearly confirmed the positive influence of wool on the vegetation of grass and the diversity of its species. Abdallah et al^63^. also confirmed the high effectiveness of wool in terms of increasing the biomass of sunflower and corn cultivated on sandy-clay soil.

On the other hand, biochar is considered to be an effective soil additive that improves the structure of soil while increasing carbon sequestration, which may eventually mitigate climate changes^64,65^. Biochar has also shown promising results in improving the yield of crops. Farhangi-Abriz et al^66^. reported that the crop yields of corn and wheat increased, respectively, by 14−35% and by 13.5% after the application of biochar on coarse-grained, acidic soils, in comparison to the control sites. This correlation was also confirmed by Khan Korai et al^67^., who noted that the yield of grain and straw in the cultivation of wheat and rice increased by 50% after the application of biochar produced from plant waste. Similarly, Munda et al^68^. demonstrated that the application of biochar from plant waste increased the yield of rice by 24%. In a comprehensive literature review, Liu et al^69^. concluded that the application of biochar increases the crops of rice on average by 11% and that it improves the effectiveness of the use of nitrogen by 12%.

Literature analysis provides several studies that indicate the colonisation of roots by *Trichoderma* fungi may improve the growth and development of roots, resistance to biotic or abiotic stress factors, and the use of nutrients^70^. The effectiveness of Trichoderma in field conditions is strongly dependent on soil properties, the presence of competing microorganisms, plant species and fungal strain^71^. The literature indicates that Trichoderma can cause strong, limited or undetectable changes in microbial communities, and this effect can vary over time^72^. The authors noted a positive influence of Trichoderma (the Rizosferin HA preparation) on the increase in the biomass of plants, but only in the first season. The presence of the preparation had only a slight influence on the increase in biomass, so it is difficult to unambiguously confirm the efficiency of this solution. According to the findings of Tyśkiewicz et al^73^. and Yao et al^74^., the potential of Trichoderma applies mainly to the protection of plants against multiple pathogens. In the present study, no pathogenic agents were introduced, which might have led to the low influence of the preparation on plant vegetation. The plants were free from any pathogens both on control sites and on those with installed composites. The potential of composites enriched with Trichoderma should be additionally verified in the presence of such pathogenic agents or with the use of other strains. Furthermore, our study results indicate that the effect of Trichoderma may be short-lived, being limited to one growing season. Such observations suggest that its efficacy may be significantly diminished once the plant growth cycle is complete, which should be taken into account when assessing the potential for the long-term use of Trichoderma in practice.

In published studies, biochar and fibres were always used separately or in combination with traditional mineral fertilization. The present study has proven that the combination of waste fibres and biochar may result on better results than applying them separately. The nonwovens used in the composite underwent gradual biodegradation. As a result of this process, nutritious substances, rich in such elements as N, P, K, and S were released into the soil. At the same time, the biochar improved water retention and reduced the washing out of nutrients, which prolonged the duration and increased the effectiveness of this solution. Even in the second vegetation season, the increase in the biomass of plants on sites equipped with composites containing biochar remained at a significantly higher level than on control sites. This indicates that the use of this solution brings better results even in the second growing season, which is difficult to achieve with biodegradable additives. Some examples are other biodegradable soil conditioners, including: geotextiles based on jute^75^, with an estimated effectiveness duration of 3 months; wool-based geotextiles^76^, with an estimated period of effective functioning of 5 months, or biodegradable SAPs based on sodium alginate with an efficiency period of 3−6 months^77^.

### Plant water management

Drought is the key factor that affects plant production and the quality and volume of crops. Water stress significantly reduces the efficiency of photosynthesis and the uptake of nutrients from soil^78^. To improve the efficiency of plant production, it is necessary to ensure that both nutrients and water will be available in soil. This requires technologies that combine the possibility to store water and nutrients. Certain soil conditioners, such as SAP, Controlled Release Fertilizers (CRF) or Slow Release Fertilizers (SRF) that provide simultaneous access to nutrients and water are available on the market^79,80^. However, their applications are limited because they are usually manufactured from synthetic materials^81^. For the purposes of this study, synthetic polymers were replaced with materials produced from waste of plant and animal origin. The biochar used in the composite had a positive influence on plant water management, which was confirmed by higher values of the RWC index observed both in the first and second vegetation season. The findings presented here are compliant with the observations of other authors. Sansan et al^82^. proved that biochar improved the moisture content in soil, which, in turn, translated into improved efficiency of the processes that protect the leaves of plants during drought. This is an important characteristic that helps minimise the negative effects of drought stress on the photosynthetic apparatus. Boudjabi et al^83^. also noted the positive influence of biochar on increasing the water reserves in leaves, even in the conditions of drought-induced stress, which was confirmed by higher values of the RWC index. The presence of biochar significantly reduced and mitigated the influence of this stressor.

In this context, it is important to emphasize that drought limits plant productivity not only by limiting water availability but also by restricting the transport and uptake of nutrients from the soil, reducing photosynthetic activity, and increasing physiological stress^84,85^. Therefore, solutions that simultaneously stabilize water and nutrient availability in the root zone are particularly effective in mitigating the effects of drought. The increased RWC values ​​observed in this study can therefore be interpreted as a physiological indicator of improved plant hydration resulting from a more stable water supply in the root zone, rather than from the short-term effects of soil moisture. The persistence of this response into the second growing season further suggests that the biochar fraction contributed to maintaining favorable soil physical conditions, even as the fibrous component of the composite gradually degraded.

The research presented here also confirmed these observations. The application of biochar in composites had a positive effect on the water content in leaves. Apart from that, the presence of nonwoven might also have contributed to the improvement of the water retention capacity of soil. Due to the fact that natural fibres have hygroscopic properties, they may absorb water during rainfall and then slowly release it to the environment in dry periods^27^. This correlation was also confirmed by Broda et al^86^. in a field experiment, that the presence of woollen nonwovens installed on a steep slope contributed to the improvement in soil water retention and helped maintain the plants in a better condition.

### Chemical composition of plants and soil parameters

N, P, and K are elements that are indispensable for optimal growth and metabolism of plants, which, in turn, translates into the volume and quality of crops^87^. Sustainable access to these elements fosters significant increase in the biomass of plants. This is compliant with the findings presented here, as on all sites equipped with biodegradable composites (rich in nutrients), the increases in the fresh and dry weight of plants were significantly higher than on control sites (without any additives). In their studies on the properties of sheep wool, Rom et al.^88^ and Broda et al.^89^ noted that wool undergoes biodegradation under the influence of enzymes released by soil microorganisms. In this process, which may take up to several months, the fibres are transformed into compounds that are rich in nutrients, such as N, C, and S. Our previous studies on BioWAG also demonstrated that the process of biodegradation of plant and animal fibres (wool, jute, and flax) lasts at least several months in actual field conditions^32,90,91^. The gradual release of nutrients throughout the vegetation season provides reasonable support for plant growth, without the need to apply additional fertilization.

The soil concentrations of nutrients on sites with installed composites remained on a significantly higher level than on control sites for two vegetation seasons. In the first vegetation season the highest content of selected nutrients was found on sites containing composites that consisted of nonwovens only (A1 and B1). On the other hand, the content of N was significantly higher on sites enriched with biochar in comparison to those that contained only nonwovens. This may result from the intensive uptake of nutrients by plants or from the adsorption of these elements by the porous structure of biochar^92^. In the second vegetation season, as a result of the progressing biodegradation of the composites, the total content of micro- and macroelements in soil was lower than in the preceding year. This is typical for solutions that undergo gradual biodegradation. However, it is worth noting that the accumulation of nutrients (N, P, K) found on sites containing biochar in the second season was significantly higher than on the remaining sites. This may be related to the very intensive growth of plants in the first vegetation season, which led to an increased uptake of nutrients from soil. In the second season, after the nonwovens had been largely decomposed, the efficiency of the composite was determined mainly by the presence of biochar. Therefore, one may conclude that biochar prolongs the effective operation of biodegradable composites.

These observations are in agreement with the findings of Banik et al^93^., who proved that using biochar in long-term applications may significantly increase the accessibility of nutrients in soil, while improving their uptake by plants (by 17−50%). Khan Korai et al^67^. noted that the addition of biochar significantly improved the accessibility of the elements N, P, and K in soil, and then increased the content of these elements in the grain (by 26 to 37%) and straw of rice and wheat (od 22 do 37%). Our results obtained in the field experiment are consistent with these trends, as plots enriched with biochar-containing composites showed an increase in plant biomass and nutrient content in both soil and plant material compared to the control, including N, P, and K^94,95^. Importantly, the persistence of the response in the second growing season suggests that the addition of biochar contributed to the stabilisation of nutrient availability over time, which is consistent with the literature indicating that biochar can reduce nutrient losses (e.g. through leaching) and improve their retention in the root zone. Moreover, the composites that consisted only of wool may have undergone biodegradation faster than their equivalents containing biochar, which may have led to a more intensive release of the elements in the first vegetation season.

It is also worth noting the long-term effects of the application of biochar. In the long-term perspective, biochar may improve water and nutrient retention and the structure of soil. In consequence, this may increase the crops and reduce external expenditures, thus contributing to saving resources and lowering production costs^96^. The high efficiency of biodegradable composites that was noted at the end of the second vegetation season may mean that they will retain their potential in the subsequent season as well.

### Synergy between fibres and biochar and practical aspects of composite application

The synergistic effect observed when using fibres and biochar can be explained based on basic biogeochemical mechanisms. Animal and plant fibres (such as wool and jute) are a source of N, P, K and S, which are gradually released into the soil as a result of biodegradation, while improving the availability of these nutrients to plants. Similar mechanisms have been observed in studies with soil additives produced from pure wool, e.g. Broda et al.^76^,, Rom et al^88^., Abdallah et al^97^., Böhme^98^, which showed that wool acts as a slow-release fertiliser that provides plants with a stable source of nutrients throughout the season. Our results are consistent with these reports, as all composite variants increased the nutrient content in soil and plant material compared to the control and simultaneously increased biomass production, indicating that nutrient release from the nonwoven fabric was effective under field conditions. Furthermore, the strongest responses in the first growing season are consistent with the expected higher intensity of fibre decomposition when the pool of easily degradable organic matter is largest^99^. In turn, biochar, thanks to its specific surface area and porous structure, increases the adsorption capacity of soils towards solutions and gases, limiting their leaching into deeper layers of the soil profile. As a result, the nutrients released during fibre decomposition remain available in the root zone for longer, which increases their efficiency of use by plants. This has been confirmed by numerous reports in the literature, which indicate that biochar increases water and nutrient retention and supports the development of plant root systems^100–102^.

Both the results of this study and reports in the literature indicate that biodegradable fibres are the main source of nutrients. However, their effectiveness when used alone is usually limited to one growing season. On the other hand, the application of biochar alone, despite its beneficial effect on soil properties, does not provide the same high efficiency in stimulating plant growth. The experiments conducted prove that the combination of both materials leads to a synergistic effect: the fibres provide easily accessible nutrients, while the biochar stabilises them in the soil profile and improves water retention. As a result, the effectiveness of biodegradable composites was maintained in the second growing season, indicating greater durability compared to solutions based solely on individual components.

## Summary and conclusions

Despite the advantages of the two-year field experiment conducted under realistic environmental conditions, the study is not without certain limitations. The experiment was carried out on a single experimental embankment located in the north-eastern part of Wrocław, Poland, within the temperate climate zone, which may have influenced the performance of the composites. Moreover, only selected grass species were included in the experiment; therefore, the observed effects of the composites are currently limited to this type of vegetation. Importantly, these limitations create valuable opportunities for future research, including field experiments conducted under diverse soil and climatic conditions, the inclusion of a broader spectrum of plant species, and more detailed investigations of soil–plant–microbial processes. Consequently, the present study should be regarded as a solid and necessary introduction to a promising technology.

Although currently used soil conditioners may be satisfactory for supporting vegetation, they are usually manufactured from non-renewable resources and may cause environmental pollution. Developing alternatives that ensure crop productivity while reducing environmental impacts remains a key challenge for sustainable agriculture.

This study demonstrated the effectiveness of biodegradable composites made from waste fibres and biochar, fully consistent with the principles of the circular economy. The innovative combination of both materials provided a synergistic effect: fibres supplied nutrients through gradual biodegradation, while biochar improved their retention and enhanced water availability.

The composites significantly increased plant growth. In the first season, fresh and dry biomass of grass increased by up to 190%, while root biomass increased by 119% compared to the control. In the second season, despite partial degradation of the fibres, biomass remained significantly higher on plots with composites, particularly those enriched with biochar. Soil fertility was also improved. Significantly higher content of nutrients (N, P, K, S, and Mn) in soil and in plants was noted on all sites with composites in comparison to the control sites. Relative water content (RWC) in leaves was also improved by up to 20%. These results indicate that the composites can sustain effectiveness for at least two vegetation seasons, outperforming many other biodegradable soil conditioners with much shorter functionality.

The proposed technology offers practical benefits for agriculture and environmental engineering by improving soil quality, reducing the need for mineral fertilizers, and enhancing water management, particularly in degraded or erosion-prone habitats. Due to the universal nature of the analysed topics, covering the global issue of waste management, supporting vegetation, and the wide range of biodegradable waste, the directions of future research may include, among others, studies on the properties of other types of waste, the optimisation of the composition of composites, and testing the effectiveness of the proposed solution in various soil and climate conditions.

## Materials and methods

### Test site and meteorological conditions

In May 2023, the experiment was started on the model embankment located in the Agricultural and Hydrological Observatory Wrocław-Swojec of the Wroclaw University of Environmental and Life Sciences (51°07′N, 17°10′E). Observations were conducted through two vegetation seasons: from May 2023 to October 2024. The test area is situated in the north-eastern part of Wrocław, located in the Silesian Lowland, in Poland, Central Europe. The Wrocław agglomeration is situated in the moderate climate zone. The average annual air temperatures in Wrocław is approx. 9.0 °C. Average annual precipitation is approx. 570 mm^103^.

**Fig. 3:**
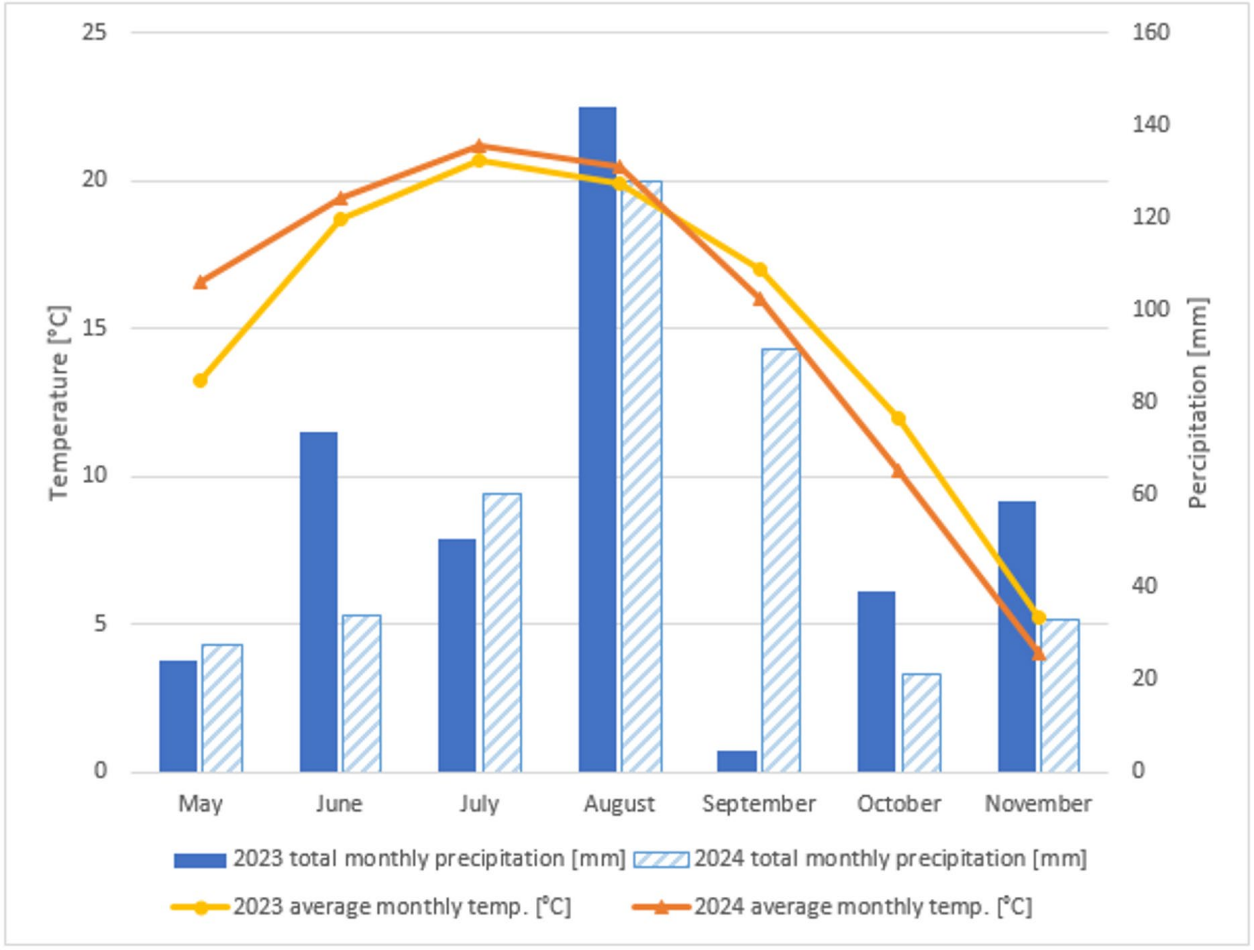
Average monthly temperatures and total precipitation noted during the field experiment in 2023–2024.

Meteorological data from the test period are presented in Fig. 3. In 2024, higher temperature values were recorded in the months of May, July and August, compared to the corresponding months of 2023. The warmest month in both years was July - with an average temperature of 20.7 °C in 2023 and 21.2 °C in 2024. The autumn drop in temperature was more pronounced in 2024, with an average temperature of just 4.0 °C in November, compared to 5.2 °C in 2023. In terms of precipitation, in 2023 the highest monthly total was recorded in August (144.1 mm). In 2024, the highest precipitation total occurred in September with 91.7 mm.

### Site description and experiment plan

The two-year field experiment was conducted on an experimental slope that had been designed and constructed in compliance with standards applicable to engineering objects including flood banks, road embankments, and slopes of reclaimed landfills. The slopes of the test sites had an inclination of 1:3. The soil embedded in the slope was classified according to the USDA Soil Classification System as loamy sand (main soil parameters: pH 6.90; P 7.42 mg·100 g^− 1^ soil; K 6.29 mg·100 g-^1^ soil; %C 1.11; %N 0.09).

The tests were conducted on seven experimental fields/blocks of the same dimensions: width of 1.5 m and length of 3.0 m. Six variants of nonwoven composites were placed on the levelled surface of the slopes and in the designated test fields. One control field (without soil additives) was left. The detailed description and presentation of the tested material is provided in sub-Sect. *2.4. Test material – nonwoven composite.* The prepared composites were in the form of mats, 1.0 m wide and 3.0 m long. Composites were placed on the experimental fields with buffer zones of the dimensions of 0.5 × 3.0 m between different variants.

The evenly placed nonwoven composites and the experimental belt were covered with an approximately 0.15 m thick layer of soil classified as loamy sand. The soil was compacted with an excavator bucket and levelled. After the earth works had been completed, the slope was sown with a mixture of grass species (the plant material is described in sub-Sect. *2.3. Characteristics of the plant material).* The experiment used a randomized block design in order to limit the influence of habitat variability on the results. The experiment was conducted in an arrangement of repetitive blocks, maintaining homogenous environmental conditions within each block (including terrain inclination, exposure, solar irradiation intensity, distribution of precipitation, and chemical and physical properties of soil).

Each block was treated as an independent experimental unit. Samples of soil and/or plants were collected randomly, with at least four iterations, from separate points within each of the blocks, to ensure that the results would be representative. Such sampling scheme allowed for the control of intra-block variability and for the improvement of the statistical power of the analyses, while minimising the influence of random factors.

The fresh and dry biomass of grasses was measured at regular intervals, on average every 8 weeks during each growing season. Three mowings were performed in each growing season. After collecting grass samples for laboratory analysis, the entire plot was mowed using a mower set to the same cutting height, which allowed for uniform regrowth of plants in all variants. During the entire experiment, no additional grass sowing was carried out, nor were any mineral or organic fertilisers or other soil additives used. Weeds were controlled exclusively by manual removal. Irrigation was carried out only immediately after sowing the seeds, until they germinated; in the following period, the only source of water was precipitation.

### Characteristics of the plant material

All sites were sown with a commercially available mixture of grass seeds, consisting of 5% *Poa pratensis;* 5% *Festuca ovina;* 20% *Festuca rubra;* and 65% *Lolium perenne*. The selected mixture of grasses is suitable for application in various habitat conditions, including in degraded areas or in difficult habitats, ensuring that the research results are more universal.

### Tested material - nonwoven composite

**Fig. 4:**
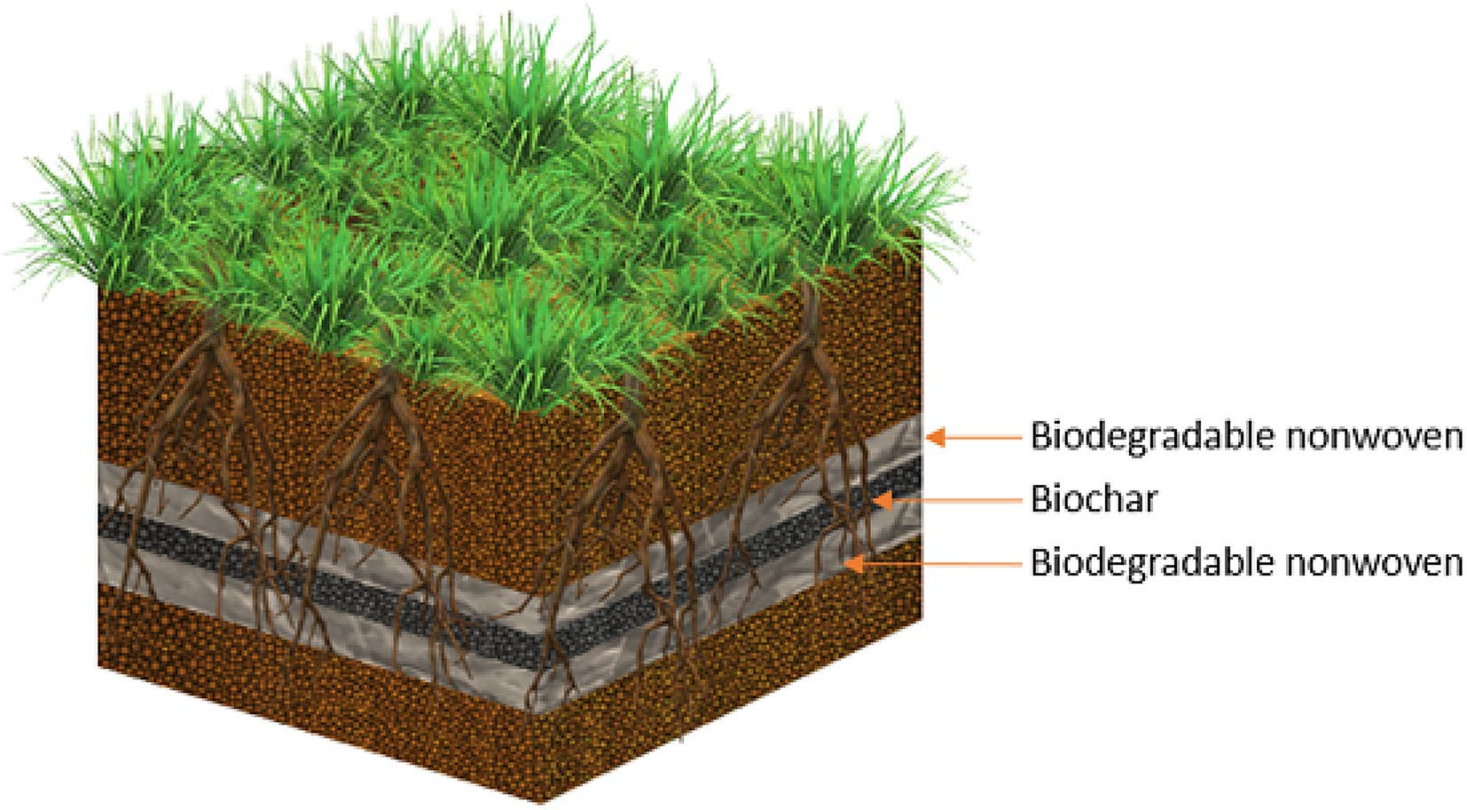
Schematic representation of an experimental setup with a composite consisting of two layers of nonwoven fabric and biochar.

To conduct a comprehensive analysis of the influence of the developed composites on the vegetation of plants in actual field conditions, several variants of the technology were applied. The variants differed in terms of the raw material composition of the nonwovens used in the production of the geocomposite, to demonstrate the potential of multiple materials. Additionally, materials with an addition of biochar were applied to prove the influence of this component on the vegetation of plants (Fig. 4). Two composite variants were additionally enriched with the biological preparation RIZOSFERIN HA, containing fungi of the Trichoderma genus, which was incorporated into the composite together with biochar to ensure uniform distribution and natural stimulation of plant growth.

Overall, six variants of nonwoven composites were used in the experiment, along with a control site:

1. Variant **A1**: Composite consisting of two layers of nonwoven (composition: 100% waste wool; surface weight of a single layer of nonwoven: 189 g∙m^−2^);
2. Variant **A1 + BIOC**: Composite consisting of two layers of nonwoven (composition: 100% waste wool; surface weight of a single layer of nonwoven: 189.0 g∙m^−2^), with biochar placed between the layers (biochar dose: 1.5 kg∙m^−2^);
3. Variant **A1 + BIOC + TRCH**: Composite consisting of two layers of nonwoven (composition: 100% waste wool; surface weight of a single layer of nonwoven: 189.0 g∙m^−2^), with biochar placed between the layers (biochar dose: 1.5 kg∙m^−2^) and RIZOSFERIN HA (dose: 15 g∙m^−2^);
4. Variant **B1**: Composite consisting of two layers of nonwoven (composition: 80% waste wool and 20% waste jute; surface weight of a single layer of nonwoven: 301.0 g∙m^−2^);
5. Variant **B1 + BIOC**: Composite consisting of two layers of nonwoven (composition: 80% waste wool and 20% waste jute; surface weight of a single layer of nonwoven: 301.0 g∙m^−2^), with biochar placed between the layers (biochar dose: 1.5 kg∙m^−2^);
6. Variant **B1 + BIOC + TRCH**: Composite consisting of two layers of nonwoven (composition: 80% waste wool and 20% waste jute; surface weight of a single layer of nonwoven: 301.0 g∙m^−2^), with biochar placed between the layers (biochar dose: 1.5 kg∙m^−2^) and RIZOSFERIN HA (dose: 15 g∙m^−2^);
7. **Control site**: without any soil additives.

#### Biochar used

The biochar used in the composite was produced from plant biomass of Giant Miscanthus. The dose of biochar was 1.5 kg∙m^− 2^ of the composite. The biochar had the following parameters: pH 8.25; C 70.90%, P 3.41 g∙kg^− 1^, N 0.27%, Mn 326.00 mg∙kg^− 1^, and K 2.35 g∙kg^− 1^^46^. The physical parameters of the biochar used were discussed extensively in the paper by Śpitalniak et al^104^.

#### Rizosferin HA used

Additionally, the commercially available preparation RIZOSFERIN HA that contains fungi from the Trichoderma genus (manufactured by TK Agronomy) was added to two variants of the nonwoven composites. The preparation consists of the following components: 15% P_2_0_5_; 3% NH_4_, and 50 g of tricho B35. The dose of the RIZOSFERIN HA preparation was 15.0 g∙m^− 2^. Trichoderma *H. asperellum*, strain B35 belongs to a cryophilic species that may be a factor for biocontrol against phytopathogens. It may also stimulate the growth of plants^104^.

The composite was manufactured with needle-punching technology. A layer of powdery biochar was placed between two layers of the nonwoven, and then the layers were joined with the use of a needle punch machine. As a result of this process, a homogeneous material was obtained in form of a composite. The needle-punch technology prevented the uncontrolled movement of the biochar and preparation RIZOSFERIN HA within the mat. Apart from that, it allowed for the production of a material of the appropriate resistance parameters that was easy to apply. To ensure the homogeneity of subsequent batches of the material, all components of the composites were precisely gauged on a laboratory scale, and then evenly distributed with the use of the needle punch machine. Subsequent batches of the material were controlled by determining their surface weight.

### Analyses of the plant and soil material

#### Analysis of the fresh and dry weight of grass

The fresh and dry weight of grass was measured, on average, every 8 weeks during each vegetation season. Grass was cut with shears in 4 randomly selected locations in each given belt/experimental field. A 30 × 50 cm frame was used. Three swathes were taken during each vegetation season. The collected plant material was transported in sealed containers to the laboratory, where the fresh weight of plants was recorded. Then the grass was dried until a fixed weight was obtained (for approximately one day) in a laboratory drier, at the temperature of 70 °C, and the dry weight was determined.

#### Relative water content (RWC)

After each recorded swathe, the relative water content (RWC) was also determined from the formula:

$$RWC=\frac{FW-DW}{TW-DW}\times100\%$$

where:

FW − fresh weight [g];

DW – dry weight [g];

TW – turgid weight [g].

#### Chemical composition of plants

Additionally, in September 2023 and in September 2024, plant material was collected to determine the content of selected macro- and microelements. Total nitrogen was measured with the use of the elemental analysis method with the Vario MACROcube apparatus (Elementar Analysensysteme GmbH, Germany). To determine the content of P, K, and Mn, the plant material was subjected to dry mineralisation, and the ash was then dissolved in nitric acid. In the solutions obtained as a result of mineralisation, the content of P was determined with the vanadium-molybdenum method, while K was determined with the flame emission spectroscopy method. The content of Mn was assayed using atomic absorption spectrometry (AAS) (Varian model SpectrAA 220FS, Varian Medical Systems, Inc., Charlottesville, VA, USA). Total S content was determined by mineralizing the plant material using the method of Butters-Chenery^103^, and then measuring turbidimetrically the amount of sulphate formed as barium sulphate as modified by Bielecki and Kulczycki^104^.

#### Root system

At the end of each vegetation season, grass root system balls were collected from each test field. They were collected from four randomly selected locations (28 samples were collected in every season, a total of 56 root system balls). Root system balls were collected from the test fields with the use of steel cylinders of the length of 0.3 m and the diameter of 0.1 m. The samples were protected by stretch foil, transported to the laboratory, and the soil was removed. The root systems were dried for approx. one day (until fixed mass was achieved) in a laboratory drier, at 70 °C. The length and weight of the dried root samples were determined, Then the Root Length Density (RLD) and Root Dry Mass Density (RDMD) were calculated.

**Root Length Density (RLD)**

$$RLD=\frac{L}{V}\left[m\bullet{m}^{-3}\right]$$.

Where:

L − total length of the root system [m].

V− soil volume [m^3^].

**Root Dry Mass Density (RDMD)**

$$RDMD=\frac{RDM}{V}\left[g\bullet{m}^{-3}\right]$$.

Where:

RDM − dry root mass [g].

V − soil volume [m^3^].

### Soil sampling and sample preparation

In September 2023 and September 2024, four soil samples were collected from three randomly selected locations on the experimental belts. The samples were collected at the depth at which nonwoven composites had been installed, i.e. 15–20 cm. Then, soil samples were transported to the laboratory. The following parameters were determined in the collected soil: soil acidity was determined in 1:2.5 soil:1 M KCl suspensions using a digital pH meter CP 505 (Elemetron Co., Zabrze, Poland). The total content of carbon and nitrogen in the samples was determined by elemental analysis using the Vario MACROcube analyser (Elementar Analysensysteme GmbH, Germany). Plant-available phosphorus and potassium levels were assessed using the Egner–Riehm DL method. Soluble manganese (Mn) content in the soil was measured with the Rinkis method, employing Atomic Absorption Spectroscopy (AAS) on a Varian SpectrAA 220FS instrument (Varian model SpectrAA 220FS, Varian Medical Systems, Inc., Charlottesville, VA, USA). Total S content was determined by mineralizing the plant material using the method of Butters-Chenery^103^ and then measuring turbidimetrically the amount of sulphate formed as barium sulphate as modified by Bielecki and Kulczycki^104^.

### Statistical analysis

Statistical evaluation of experimental data employed analysis of variance (ANOVA) with single-factor design. Prior to variance analysis, data sets underwent validation for normality using the Shapiro–Wilk normality test, while variance homogeneity across treatment groups was assessed through Levene’s test. Post-hoc multiple comparisons between treatment means were conducted using Tukey’s HSD (Honestly Significant Difference) procedure, with statistical significance set at α = 0.05. Data processing and statistical computations were executed in R statistical environment (version 4.4.0, R Foundation for Statistical Computing, Vienna, Austria).

## Data Availability

Data will be made available on request. The data that support the findings of this study are available from the corresponding author, [D.M.], upon reasonable request.
